# Botryoid embryonal rhabdomyosarcoma of the cervix: A case report

**DOI:** 10.1016/j.ijscr.2022.107858

**Published:** 2022-12-31

**Authors:** Alejandro Delfos Hermoza, Graziela de Macêdo Matsushita, Marcelo Henrique dos Santos, Ronaldo Luis Schmidt, Ricardo dos Reis, Carlos Eduardo Mattos da Cunha Andrade

**Affiliations:** a Fellow of Oncology Surgery, Barretos Cancer Hospital, Barretos, Brazil; b Department of Pathology, Barretos Cancer Hospital, Barretos, Brazil; c Department of Gynecologic Oncology, Barretos Cancer Hospital, Barretos, Brazil

**Keywords:** Embryonal rhabdomyosarcoma, Botryoid, Cervix, Surgery, Chemotherapy

## Abstract

**Introduction and importance:**

Rhabdomyosarcoma (RMS) is a malignant tumor that arises from embryonal skeletal muscle cells. It's responsible for 3 % of cancer cases among children aged from 0 to 14 and 1 % among adolescents and young adults aged from 15 to 19. Embryonal RMS (ERMS) is the most prevalent subtype of rhabdomyosarcoma in the female genital tract. Botryoid sarcomas are a polypoid variant of ERMS. Our objective is to describe the clinical, pathological features and the treatment of a patient diagnosed with RMS botryoid of the cervix.

**Case presentation:**

We report a case of a 19-year-old female patient diagnosed with botryoid RMS of the cervix. The histopathological evaluation of the cervix showed a polypoid tumor lined by squamous epithelium exhibiting a large hypocellular edematous area. It was classified as group II and stage 1, according to the IRSG multicenter studies. Cervical polypectomy was performed as an oncological surgical treatment and adjuvant chemotherapy consisting of Vincristine 1.5 mg/m2/day and Actinomycin D 0.045 mg/kg/day (VA) for 45 weeks. After 6 months of follow up, she had no evidence of recurrence.

**Clinical discussion:**

Cervical ERMS is a rare tumor, especially in adolescence. It's usually presents as a cervical polyp or multiple polyps. Multimodal approaches have remarkably improved the prognosis and decreased the need for radical surgery with its associated morbidity.

**Conclusion:**

There are a variety of treatment strategies for a rare disease such as cervical botryoid RMS. This case was approached through fertility-conserving surgery, followed by adjuvant chemotherapy and oncological clinical follow up.

## Highlights

• Rhabdomyosarcoma is a malignant tumor that arises from embryonal skeletal muscle cells.
• Botryoid sarcomas are a rare polypoid variant of embryonal rhabdomyosarcoma.
• Multimodal approaches improve the prognosis and decreased the need for radical surgery.
• Fertility-conserving surgeries in the early stages of the disease are allowed.

## 1 Introduction

Rhabdomyosarcoma (RMS) is a malignant tumor that arises from embryonal skeletal muscle cells [1]. It is the most common soft tissue sarcoma of childhood, accounting for 3 % of cancer cases among children aged from 0 to 14 and 1 % of cases among adolescents and young adults aged from 15 to 19. The 5-year survival ranges from 70 % for children to 50 % for adolescents and young adults [2].

Embryonal RMS (ERMS) is the most prevalent subtype of RMS in the female genital tract. Predominately arising from the vagina during childhood. In 36 % of cases it occurs before the age of 5 and, uncommonly, below 1. In adolescents and postmenopausal women, the most frequent place is in the cervix and uterine corpus [3]. There are several histological subtypes of RMS: ERMS, botryoid ERMS, spindle cell variants of ERMS, and alveolar RMS (ARMS). ERMS is the most common subtype and approximately three times more frequent than botryoid ERMS and ARMS [4].

Botryoid sarcomas are a polypoid variant of embryonal RMS, arising from embryonal rhabdomyoblasts and constituting approximately 3 % of all RMSs [5]. There is no standard treatment for patients with cervical RMS, although most patients are treated with surgery, chemotherapy and radiotherapy [1]. Our objective is to describe the clinical, pathological features and the treatment of a patient diagnosed with cervical botryoid RMS. This case was reported according to the SCARE 2020 criteria [6].

## 2 Case presentation

A 19-year-old patient presented a 2-month history of a painless and fibroelastic vaginal mass of approximately 3 cm, arising from the cervix, mobile during urination and without vaginal bleeding. No surgical history, drug history, family history, including any relevant genetic information, and psychosocial history.

Polypectomy of the cervix was performed with sampling of two cervical polyps of approximately 4 × 5 cm and 3 × 2 cm by hysteroscopy at the hospital of origin by a gynecologist. A histopathological slide review was performed at our institution, confirming the diagnosis of botryoid RMS with compromised margins.

**Fig. 1:**
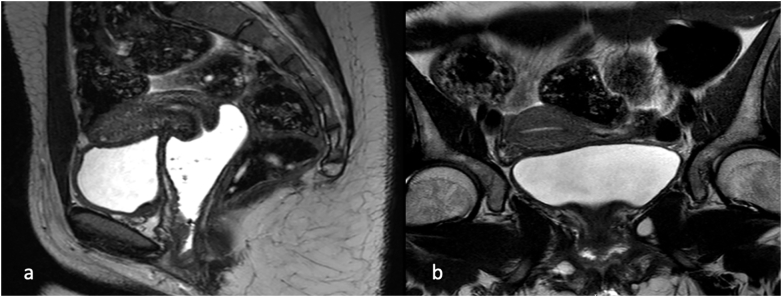
(a-b) MRI of the pelvis without evidence of oncologic disease.

Oncological staging, including magnetic resonance imaging of the pelvis (Fig. 1), computed tomography of the chest and abdomen, did not reveal local or distant disease. Multiple cervical and endometrial biopsies were performed without evidence of oncological disease.

**Fig. 2:**
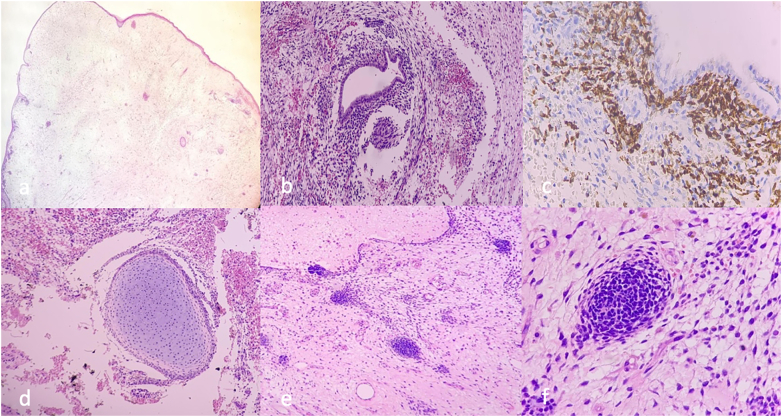
(a) Polypoid tumor lined by squamous epithelium showing a large hypocellular edematous area. (b) Benign gland surrounded by a hypercellular stroma composed of small, round and blue cells with scant cytoplasm. (c) Positive for the Desmin immunohistochemical marker. (d) Cartilaginous heterologous element among of the hypocellular and hemorrhagic stroma. (e-f) Cohesive nests composed of small, round and blue cells among of edematous, hypocellular stroma. (For interpretation of the references to colour in this figure legend, the reader is referred to the web version of this article.)

The cervix histopathological evaluation showed a polypoid tumor lined by squamous epithelium exhibiting a large hypocellular edematous area (Fig. 2a). Tumor cells surrounded by a hypercellular stroma composed of small, round and blue cells with scant cytoplasm (Fig. 2b). A heterologous cartilaginous element was observed among of the hypocellular and hemorrhagic stroma (Fig. 2d) and cohesive nests composed of small, round and blue cells among the edematous and hypocellular stroma (Fig. 2e).

**Fig. 3:**
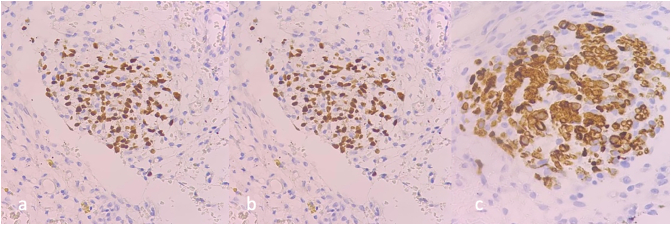
The cells that make up the cohesive nests are positive for the following immunohistochemical markers: (a) Myogenin, (b) MyoD1 and (c) Desmin.

The cells that make up the cohesive nests were positive by immunohistochemistry, performing the Desmin, Myogenin, HHF35 and Myo-D1 markers (Fig. 3).

After a multidisciplinary discussion between oncology surgeons, clinical oncologists, pediatricians, radiologists and pathologists, the patient with a diagnosis of localized tumor, non-metastatic RMS, microscopically involved margins, was classified as group II and stage 1, according to the IRSG multicenter studies [4]. She agreed about the treatment that was going to be offered. Underwent adjuvant chemotherapy treatment consisting of Vincristine 1.5 mg/m^2^/day and Actinomycin D 0.045 mg/kg/day (VA) for 45 weeks. She had a good adherence and tolerability to the chemotherapy intervention. Patient maintaining relative rest at home with scheduled outpatient appointments. After 6 months of follow up, she had no evidence of recurrence. The surveillances plans are hysteroscopy and computed tomography scan of the pelvis every 6 months for the first 2 years.

## 3 Discussion

Cervical ERMS is a rare tumor, especially in adolescence. It usually presents as a cervical polyp or multiple polyps [7], [8]. The botryoid subtype of embryonal RMS accounts for about 10 % of all cases of RMS and appears on the mucosal surface of hollow viscera such as the vagina, bladder, and cervix. It predominates in children and young adults. Most patients experience vaginal bleeding or a mass sensation at the introitus. Our patient falls into this age group for botryoid embryonal RMS of the cervix and presented with a vaginal mass, arising from the cervix not associated with vaginal bleeding [1], [9].

The diagnosis is made by clinical findings and confirmed by histology, as was the case of this patient who had a vaginal mass resection, and the diagnosis was confirmed by pathological anatomy [7].

Histologically, they are polypoid tumors, characterized by a proliferation of edematous hypocellular spindle cells, with cell condensation under the epithelial surfaces, classified as botryoid. The characteristics expressed in the immunohistochemical were Desmin and Myogenin [10].

Multimodal approaches have remarkably improved the prognosis and decreased the need for radical surgery with its associated morbidity. The appropriate treatment for cervical botryoid sarcoma is still debatable. Surgical procedures include simple hysterectomies, radical hysterectomies with or without lymphadenectomy, and more conservative procedures such as polypectomies or local excisions [11], [12].

Copeland et al. performed one of the largest series of cases of botryoid RMS of the female genital tract, that showed the evolution of treatment over a period of 30 years [13]. On the other hand, Daya et al. were the first to demonstrate a conservative approach to these tumors with favorable evolution [11]. The IRSG modified the treatment protocols combining chemotherapy and radiotherapy surgery, improving the prognosis and survival of these patients, allowing more fertility-conserving surgeries in the early stages of the disease. The treatment prescribed on the basis of this study defines the groups by the extent of the disease and by the completeness or extent of the initial surgical resection after pathological review of the tumor characteristics [4].

## 4 Conclusion

There is a variety of treatment strategies for a rare disease such as cervical botryoid RMS and are built on the experience of multiple studies. This case was approached through fertility-conserving surgery, followed by adjuvant chemotherapy and oncological clinical follow up.

## Provenance and peer review

Non-commissioned, external peer-reviewed.

## Consent

Written informed consent was obtained from the patient for publication of this case report and accompanying images. A copy of the written consent is available for review by the Editor-in-Chief of this journal on request.

## Ethical approval

None.

## Funding

None.

## Guarantor

Alejandro Delfos Hermoza.

Carlos Eduardo Mattos da Cunha Andrade, MD.

## Research registration number

None.

## CRediT authorship contribution statement

**Alejandro Delfos Hermoza:** Conceptualization, Methodology, Writing – original draft. **Graziela de Macêdo Matsushita:** Methodology, Supervision. **Marcelo Henrique dos Santos:** Methodology, Supervision. **Ronaldo Luis Schmidt:** Methodology, Supervision. **Ricardo dos Reis:** Conceptualization, Methodology, Supervision, Writing – review & editing. **Carlos Eduardo Mattos da Cunha Andrade:** Conceptualization, Methodology, Supervision, Writing – review & editing.

## Declaration of competing interest

The authors declare that they have no known competing financial interests or personal relationships that could have appeared to influence the work reported in this paper.
